# Ketamine, Dissociation, and Depression: What Is “Special” About Ketamine? (Revisited)

**DOI:** 10.1093/ijnp/pyae024

**Published:** 2024-06-12

**Authors:** Mina M Rizk, James W Murrough

**Affiliations:** Depression and Anxiety Center for Discovery and Treatment, Department of Psychiatry, Icahn School of Medicine at Mount Sinai, New York, New York, USA; Depression and Anxiety Center for Discovery and Treatment, Department of Psychiatry, Icahn School of Medicine at Mount Sinai, New York, New York, USA; VISN 2 Mental Illness Research, Education, and Clinical Center (MIRECC), James J. Peters VA Medical Center, Bronx, New York, USA; Department of Neuroscience, Icahn School of Medicine at Mount Sinai, New York, New York, USA

In this issue of the Journal, Sajid and colleagues report on the relationship between acute dissociative effects of ketamine and changes in depression severity or suicidal thinking at subsequent timepoints in individuals with major depressive disorder (MDD). This study represents a secondary analysis derived from the ketamine arm (n = 40) of a randomized controlled clinical trial involving n = 80 adults treated with a single fixed dose of ketamine delivered via an i.v. route at a dose of 0.5 mg/kg infused over 40 minutes compared with the benzodiazepine midazolam delivered i.v. at a dose of 0.02 mg/kg over 40 minutes (Grunebaum et al., 2018). Using the standard Clinician-Administered Dissociative States Scale (CADSS) (Bremner et al., 1998), dissociation was measured at baseline, 40 minutes, 230 minutes, and 1 day post-treatment, with 40 minutes corresponding to the anticipated peak dissociative effect of ketamine. The primary finding of the current report is a lack of association between the acute dissociate effect of ketamine and a subsequent change in depression severity or suicidal thinking (at either 230 minutes or 1 day post-treatment) measured using the Hamilton Depression Rating Scale and Scale for Suicidal Ideation, respectively. Interestingly, at the 230-minute timepoint, less of an increase in CADSS score was associated with better effects on mood assessed by Profile of Moods Scale. The authors also explored relationships between dissociation and individual metabolites of ketamine at various timepoints, but the results were inconclusive. The limitations of this study include small sample size, its post-hoc nature, and lack of control for multiplicity. Be that as it may, in this well-controlled study, there was a clear lack of evidence to support the hypothesis that the acute dissociative effect of ketamine is related to its therapeutic benefit. In this commentary, we will briefly discuss a few potential implications of this observation.

The phencyclidine derivative ketamine was developed in the 1960s as an anesthetic agent capable of producing rapid anesthesia and analgesia at doses between 1.0 and 2.0 mg with minimal respiratory depression or tachyphylaxis upon repeated dosing. At doses below the threshold for loss of consciousness, individuals in early clinical trials of ketamine were noted to experience floating sensations and alternated sensory experiences involving their bodily sensations, such as being unable to feel their arms or legs. With little likeness to the effects of other agents (apart from phencyclidine), early researchers struggled to describe and name these unusual effects of ketamine, eventually coalescing around the concept of an engendered state of “disconnection” and a description of ketamine as a “dissociative anesthetic” (Domino, 2010). Possessing a complex pharmacology featuring high affinity, noncompetitive blockade of the glutamate N-methyl-d-aspartate receptor, but with minimal direct effect on monoamine signaling, early reports of a rapid-onset antidepressant effect of ketamine in individuals with MDD (Berman et al., 2000), and then treatment-resistant depression (TRD) (Zarate et al., 2006; aan het Rot et al., 2010; Murrough and Charney, 2010; Murrough et al., 2013b, 2013a), were met with justified skepticism. No agent had previously demonstrated a therapeutic effect for depression on the timescale of ketamine, nor had an agent with such a different pharmacology from the traditional oral monoaminergic antidepressants shown robust, replicable antidepressant action. Although the early antidepressant effects of ketamine have been confirmed through subsequent research, and the S-enantiomer of the racemate (“esketamine”) has gained regulatory approval for the treatment of TRD in the United States, the European Union, and elsewhere, the question remains today: What is “special” about ketamine?

The question as to the role, or necessity, of the unusual acute experience that ketamine engenders to the therapeutic benefit of ketamine has been ever-present in the field since the early studies of ketamine for depression. The answer to this question has major implications for our understanding of the mechanisms of depression and the advancement of novel therapeutics for the illness. For example, the discovery of the antidepressant effects of ketamine prompted a significant and ongoing investment in the drug discovery industry to develop compounds that would replicate or improve on ketamine’s therapeutic effects but would be devoid of its acute dissociative “side effects.” While these efforts are ongoing, it is notable that to date no compound has advanced to market as a result of these efforts (Murrough et al., 2017). If dissociative or other acute mind-altering effects of ketamine are instrumental—or inherently inseparable—from its antidepressant action, of course the efforts to divorce these effects are doomed from the start. If the effects cannot be separated, there are 2 broad, non-exclusionary possibilities as to why this would be the case. From a pharmacological perspective, it may simply be that the biological pathways that are engaged as a necessary condition of ketamine’s antidepressant effects are partially or completely overlapping with those that give rise to the dissociation such that separating them is either impossible or impractical. From a psychological perspective, it may be that the experience of “disconnection” allows for a unique rearrangement or processing of psychological and emotional material in a short timeframe that in fact is essential to the therapeutic benefit of ketamine.

The current report by Sajid et al. is consistent with other studies in the literature that find a lack of association between the acute dissociate experience of ketamine and its therapeutic benefit. For example, we analyzed a case series of 205 i.v. ketamine infusions in 97 adult participants with MDD and found that changes in CADSS score did not differ significantly between ketamine responders and nonresponders (Wan et al., 2015). Other groups have reported similar findings (Carlson et al., 2013; Shiroma et al., 2014; Wilkinson et al., 2018; Fava et al., 2020). In the larger datasets generated from the FDA registration trials of esketamine, the authors likewise report little to no correlation between acute dissociation and longer-term antidepressant benefit (Popova et al., 2019; Chen et al., 2022; Mathai et al., 2023). So, the jury is in? Maybe, maybe not. Several other studies in literature do find a partial association between dissociation and benefit following ketamine. For example, the Zarate group has found evidence for an association in a mixed sample of individuals with MDD as well as bipolar depression (Luckenbaugh et al., 2014; Niciu et al., 2018). In a separate study involving 41 patients with TRD, Phillips et al. (2019) found that the increase in the CADSS total score 40 minutes after ketamine administration correlated with depression severity reduction at 24 hours post-infusion. Other studies in literature not summarized here contribute to a mixed picture.

The question concerning the role of an acute altered state in ketamine’s therapeutic mechanism of action has taken on new light in the wake of the resurgence of interest and research on the potential of psychedelic agents in psychiatry. Classic serotonergic psychedelics—such as psilocybin, Ayahuasca (a brew containing N,N-dimethyltryptamine and harmine), and lysergic acid diethylamide—have emerged as candidate therapeutics for depression and share a characteristic feature of engendering a transient mind-altering experience followed by a potential lifting of depression, which is variably durable. While not considered a classic psychedelic, ketamine does share some of these features. The primary proximal biological mechanism of ketamine is thought to be modulation of glutamate via N-methyl-d-aspartate receptor antagonism, whereas classic psychedelics primarily enhance serotonergic signaling via agonism at the 5-hydroxytryptamine receptor 2A receptor and binding to other serotonergic receptors (Johnston et al., 2023). Despite these differences in their primary mechanisms of action, both classes of drugs appear to produce rapid and sustained antidepressant effects after a transient psychoactive period (Kadriu et al., 2021), acknowledging that the evidence base for the classic psychedelics is substantially smaller than that for ketamine at present. Basic work in animals does suggest at least a partial convergence of ketamine and classic serotonergic psychedelics on downstream pathways that positively regulate neuroplasticity relevant to their therapeutic action (Ly et al., 2018; Moda-Sava et al., 2019). An interesting wrinkle is that recent preclinical work suggests that, although acute 5-hydroxytryptamine receptor 2A receptor agonism is very likely to be the principal cause of the acute psychedelic experience, it may not be the mechanism underlying its antidepressant effects (Hesselgrave et al., 2021). This then raises the possibility that while it is generally assumed that the acute mind-altering effect of these agents (and the psychological processing that follows) are instrumental to their therapeutic benefit, in fact these processes may be separated and that the latter not dependent on the former.

Inspired by the resurgence in psychedelic medicine, some clinicians use the dissociative effects of ketamine to introduce various psychotherapeutic approaches. Ketamine is reported to foster openness and promote introspection in some individuals and settings, potentially facilitating more impactful engagement in psychotherapy during or after treatment (Dore et al., 2019). Despite the variance in how ketamine-assisted psychotherapy is applied, early reports suggest that it can prolong clinically significant reductions in pain, anxiety, and depressive symptoms (Drozdz et al., 2022). Future, well-controlled studies of ketamine-assisted psychotherapy will be needed to understand the relative risks and benefits of this approach in contrast to other potential therapy options in MDD or TRD, including ketamine alone.

## Interest Statement

In the past 24 months, Dr Murrough has provided consultation services and/or served on advisory boards for LivaNova, KetaMed, Inc, Merk, Cliniclabs, Inc., Biohaven Pharmaceuticals, Inc., Compass Pathfinder, Xenon Pharmaceuticals, and Clexio Biosciences. Dr Murrough is named on a patent pending for neuropeptide Y as a treatment for mood and anxiety disorders and on a patent pending for the use of KCNQ channel openers to treat depression and related conditions. The Icahn School of Medicine (employer of Dr Murrough) is named on a patent and has entered into a licensing agreement and will receive payments related to the use of ketamine or esketamine for the treatment of depression. The Icahn School of Medicine is also named on a patent related to the use of ketamine for the treatment of PTSD. Dr Murrough is not named on these patents and will not receive any payments. Dr Rizk declares no competing interests.
